# The impact of H63D HFE gene carriage on hemoglobin and iron status in children

**DOI:** 10.1007/s00277-016-2792-x

**Published:** 2016-08-24

**Authors:** Kaczorowska-Hac Barbara, Luszczyk Marcin, Antosiewicz Jedrzej, Ziolkowski Wieslaw, Adamkiewicz-Drozynska Elzbieta, Mysliwiec Malgorzata, Milosz Ewa, Kaczor Jan Jacek

**Affiliations:** 1 Department of Occupational Therapy, Gdansk University of Physical Education and Sport, K Gorski 1 Str., 80-336 Gdansk, Poland; 2 Department of Physiology, Gdansk University of Physical Education and Sport, Gdansk, Poland; 3 Department of Bioenergetics and Physiology of Exercise, Medical University of Gdansk, Gdansk, Poland; 4 Department of Bioenergetics and Nutrition, Gdansk University of Physical Education and Sport, Gdansk, Poland; 5 Department of Pediatrics, Hematology and Oncology, Medical University of Gdansk, Gdansk, Poland; 6 Department of Pediatrics, Diabetology and Endocrinology, Medical University of Gdansk, Gdansk, Poland; 7 Laboratory of Molecular Biology at the Medical University of Gdansk, Gdansk, Poland; 8 Department of Physiotherapy, Gdansk University of Physical Education and Sport, Gdansk, Poland

**Keywords:** Children, HFE mutation, Iron, Ferritin, Transferrin saturation, Hemoglobin

## Abstract

The molecular mechanism that regulates iron homeostasis is based on a network of signals, which reflect on the iron requirements of the body. Hereditary hemochromatosis is a heterogenic metabolic syndrome which is due to unchecked transfer of iron into the bloodstream and its toxic effects on parenchymatous organs. It is caused by the mutation of genes that encode proteins that help hepcidin to monitor serum iron. These proteins include the human hemochromatosis protein -HFE, transferrin-receptor 2, hemojuvelin in rare instances, and ferroportin. HFE-related hemochromatosis is the most frequent form of the disease. Interestingly, the low penetrance of polymorphic HFE genes results in rare clinical presentation of the disease, predominantly in middle-aged males. Taking into account the wide dispersion of HFE mutation in our population and also its unknown role in heterozygotes, we analyzed the impact of H63D HFE carriage in the developmental age, with respect to gender, on the iron status and hemoglobin concentration of carriers in comparison to those of wild-type HFE gene (12.7 ± 3.07 years, 42 boys and 41 girls). H63D carriers presented higher blood iron, transferrin saturation, and ferritin concentration than wild-type probands (*p* < 0.05.) Interestingly, male H63D carriers showed higher hemoglobin concentration than the unburdened children. Moreover, in the H63D carrier group, a positive correlation between iron and hemoglobin was noted. In conclusion, this study demonstrates that changes in iron metabolism occur at a young age in HFE heterozygotes.

## Introduction

Hereditary hemochromatosis is an autosomal recessive disorder associated with increased intestinal absorption of dietary iron and deposition of excessive amounts of iron in the liver, pancreas, and other organs [1, 2]. The syndrome is a result of genetically determined failure to stop iron entering the circulatory pool when it is not needed. It is associated with several pathogenic mutations of at least five genes (HFE, TfR2, HJV, HAMP, and SLC40A1), and it is likely due to a regulatory defect in iron homeostasis [3, 4]. HFE-related hemochromatosis is the most common genetic disorder in northern European populations with a prevalence of 1:200 to 1:250 for homozygosity and a carrier rate of 1:8 to 1:12 [5, 6]. C282Y polymorphism of the HFE gene is particularly associated with disease development. In the abovementioned populations, simple H63D heterozygosity occurs in at least 23.6–31.1 % [7]. Hereditary hemochromatosis is characterized by enhanced intestinal absorption of iron and its excess accumulation resulting in multiple organ damage, such as cirrhosis, hepatoma, diabetes mellitus, arthritis, and cardiomyopathy. The symptomatic phenotype preceded by fatigue, arthropathy, and impotence occurs predominantly in males between the fourth and sixth decades of life, depending on gene penetration and multiple concomitant factors in C282Y homozygotes, in some cases H63D/C282Y compound heterozygotes, and occasionally H63D homozygotes [1, 8]. HFE gene mutation impact on iron accumulation during the clinical course of hemochromatosis is unpredictable due to reduced gene penetration and coincidence of concomitant endogenic, environmental, and other unknown factors [9–12]. However, HFE homozygotic and even heterozygotic genotypes might be associated with disease progression in the presence of concomitant diseases. The clinical impact of HFE mutation per se still remains unknown. Notably, since iron accumulation is a prolonged process, iron overload is rarely observed in children [13]. Therefore, the developmental population seems to be a promising group to make preliminary observations considering possible HFE gene impact on future iron storage. In our presented investigations, we compared iron metabolism in the developmental age, with respect to gender, of H63D carriers with that of the wild-type group. Moreover, taking into consideration the role of iron in hematopoiesis, we analyzed hemoglobin concentration in particular groups. Iron, ferritin concentration, and transferrin saturation were statistically higher in both H63D gender groups. Moreover, hemoglobin concentration was statistically higher in male H63D carriers than in other groups. Remarkably, the H63D group presented with a statistically significant positive correlation between iron and hemoglobin concentration.

## Material and methods

Eighty-three, i.e., nonkindred children of Polish origin aged 12.7 ± 3.07 (42 boys and 41 girls), were considered for the study. Fifty children, aged 12.6 ± 2.41 (25 boys and 25 girls) presented with wild-type HFE gene, whereas 33 children, aged 12.8 ± 3.95 (17 boys and 16 girls), were carriers of HFE H63D mutation (data presented in Table 1). Patients’ BMI did not differ statistically. In order to exclude acute and chronic illnesses, or internal organ insufficiency, all patients underwent physical examination, laboratory assays which included a full blood count with reticulocytosis and microscopic evaluation, protein C concentration, aspartate and alaninie transaminase activities, bilirubin, creatinine levels, HBsAg, and anti-HCV antibodies. Iron metabolism was also assessed by measuring iron concentration, ferritin levels, and transferrin saturation (SYSMEX XE 2100, Architect ci 8200, and Test 1 SDL). Abdominal ultrasound was also performed and did not reveal any abnormalities. Patients underwent genetic testing for HFE mutations (H63D S65C C282Y), which was performed using real-time PCR. The study was officially approved by the Bioethical Committee of the Regional Medical Society in Gdansk NKBBN/409/2013 in accord with the Helsinki Declaration.

**Table 1:** Anthropometric characteristics of the subjects

Mutation		Age (years)	Weight (kg)	Height (cm)	BMI (kg/m^2^)
Wild type	F (*n* = 25)	12.1 ± 2.62	42.1 ± 14.1	149.8 ± 3.3	18.3 ± 3.63
	M (*n* = 25)	13.2 ± 2.11	51.3 ± 16.1	159.8 ± 15.9	19.6 ± 3.19
	All (*n* = 50)	12.6 ± 2.41	46.7 ± 15.7	154.8 ± 15.3	19.0 ± 3.45
H63D	F (*n* = 16)	12.0 ± 4.43	40.6 ± 20.8^a^	147.1 ± 22.5^c^	17.6 ± 5.26
	M (*n* = 17)	13.6 ± 3.39	57.8 ± 19.8^b^	164.8 ± 20.5	20.4 ± 4.11
	All (*n* = 33)	12.8 ± 3.95	49.5 ± 21.8	156.2 ± 23.0	19.1 ± 4.84

Results are shown as means ± SD

*F* female, *M* male, *n* numbers, *BMI* body mass index

Statistical significant difference:

Weight; ^a^H63D_F/H63D_M, *p* = 0.0306; ^b^H63D_M/WT_F, *p* = 0.0468

Height; ^c^H63D_F/H63D_M, *p* = 0.0288

### Statistical analysis

Statistical analysis of the data included Student’s *t* tests for independent samples to compare categorical variables between wild-type and H63D mutation groups. Differences between gender subgroups of wild-type and H63D mutation groups were calculated with nonparametric Kruskal-Wallis unpaired test. Correlations between iron concentration and hemoglobin were examined by Pearson’s correlation coefficient. Values of *p* ≤0.05 were considered statistically significant. Calculations were performed using Statistica 10.0 (StatSoft 2011) (References StatSoft, Inc. (2011); STATISTICA (data analysis software system), version 10; www.statsoft.com).

## Results

**Fig. 1:**
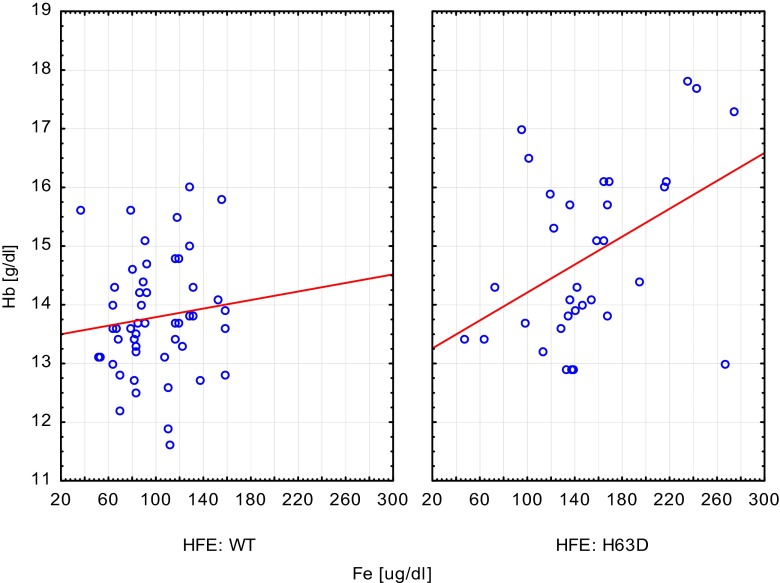
Correlation between iron and hemoglobin concentration. H63D group presented with statistical significant positive correlation. Wild-type group (WT): *y* = 13.4254 + 0.0036*x; *r* = 0.1125; *p* = 0.4368; *r* ^2^ = 0.0126. Carriers group (H63D): *y* = 13.0201 + 0.0119*x; *r* = 0.4382; *p* = 0.0107; *r* ^2^ = 0.1920

**Fig. 2:**
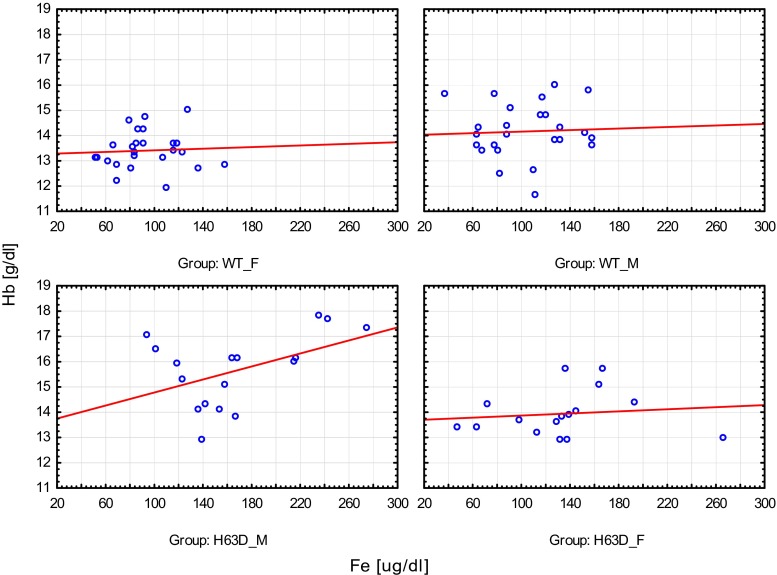
Correlation between iron and hemoglobin concentration in gender groups. There were no statistical differences. Wild-type female group (WT_F): *y* = 13.2572 + 0.0016*x; *r* = 0.0580; *p* = 0.7832; *r* ^2^ = 0.0034. Wild-type male group (WT_M): *y* = 14.0069 + 0.0015*x; *r* = 0.0473; *p* = 0.8225; *r* ^2^ = 0.0022. Carriers male group (H63D_M): *y* = 13.4959 + 0.0128*x; *r* = 0.4658; *p* = 0.0595; *r* ^2^ = 0.2170. Carriers female group (H63D_F): *y* = 13.6595 + 0.0021*x; *r* = 0.1208; *p* = 0.6559; *r* ^2^ = 0.0146

In the entire group of tested children, the mean concentration of iron, ferritin, and transferrin saturation were within normal range (mean values: iron 119.9 μg/dl, SD 48.8; ferritin 32.0 ng/ml, SD 19.0; TIBC 224.9 μg/dl, SD 77.4; and transferrin saturation Ts 32.6 %, SD 13.7). The wild-type HFE gene group (WT group) presented with a mean iron concentration of 99.1 μg/dl, SD 30.7; ferritin 28.3 ng/ml, SD 15.7; TIBC 273.4 μg/dl, SD 45.6; and transferrin saturation 27.1 %, SD 8.3. The H63D mutation carrier group presented with a mean iron concentration of 151.5 μg/dl, SD 54.3; ferritin concentration 37.6 ng/ml, SD 22.1; TIBC 151.5 μg/dl, SD 54.3; and transferrin saturation of 40.9 %, SD 16.1. All the values were statistically higher in the entire H63D carrier group, compared to the wild-type group except for TIBC which was statistically lower (data presented in Table 2). Interestingly, hemoglobin concentration and hematocrit levels of the entire H63D carrier group was statistically higher than that of the wild type, while a similar relation was not observed in red blood cell number. Notably, there were some other phenomena noted regarding gender groups such as male H63D carriers having statistically higher iron concentration than female H63D carriers and both WT males, and females as well. In addition, female H63D carriers had statistically higher iron concentration than WT females. Male H63D carriers presented with a statistically lower TIBC and a statistically higher transferrin saturation than both WT gender groups. Female H63D carriers had statistically lower TIBC than both WT gender groups. Hemoglobin concentration of male H63D carriers was statistically higher than that of the other groups. Male H63D carriers presented with a higher red blood cell count than both female groups and higher hematocrit levels than WT female group (data presented in Table 3). Statistically significant positive correlation between iron and hemoglobin concentration was noted in the entire H63D group compared with the healthy group, whereas with respect to gender groups (male and female H63D carriers compared to healthy controls), such phenomenon was not noted (data presented in Figs. 1 and 2, Tables 4 and 5).

**Table 2:** Iron status parameters in control and H63D mutation group

Variables	All (*n* = 83)	WT (*n* = 50)	H63D (*n* = 33)	*p* value
Fe (μg/dl)	119.9 ± 48.8	99.1 ± 30.7	151.5 ± 54.3	0.0000^a^
Ferritin (ng/ml)	32.0 ± 19.0	28.3 ± 15.7	37.6 ± 22.1	0.0425^a^
Ts (%)	32.6 ± 13.7	27.1 ± 8.31	40.9 ± 16.1	0.0000^a^
Hb (g/dl)	14.2 ± 1.30	13.8 ± 0.99	14.8 ± 1.47	0.0018^a^
RBC (ml/dl)	4.93 ± 0.45	4.86 ± 0.39	5.02 ± 0.52	0.1305
Ht (%)	42.1 ± 5.70	40.5 ± 2.76	44.4 ± 7.90	0.0015^a^
TIBC	224.9 ± 77.4	273.4 ± 45.6	151.5 ± 54.3	0.0000^a^

Results are shown as means ± SD

*WT* wild type, *Fe* iron, *Ts* transferrin saturation, *Hb* hemoglobin concentration, *RBC* red blood cells, *Ht* hematocrit, *TIBC* total iron-binding capacity

^a^Statistical significant difference

**Table 3:** Iron status parameters in female and male of control and H63D mutation groups

Variables	WT_F (*n* = 21)	WT_M (*n* = 39)	H63D_F (*n* = 27)	H63D_M (*n* = 31)
Fe (ug/dl)	93.9 ± 26.8	104.4 ± 33.9	134.0 ± 52.6^a^	167.9 ± 52.0^b,c^
Ferritin (ng/ml)	28.7 ± 16.2	27.8 ± 15.6	32.7 ± 19.8	42.1 ± 23.7
Ts (%)	27.1 ± 7.83	27.0 ± 8.93	36.8 ± 15.0	44.7 ± 16.6^b,c^
Hb (g/dl)	13.4 ± 0.74	14.2 ± 1.08	13.9 ± 0.90^d^	15.7 ± 1.43^b,c^
RBC (ml/dl)	4.68 ± 0.35	5.05 ± 0.33^e^	4.73 ± 0.47^d^	5.30 ± 0.41^b^
Ht (%)	39.6 ± 2.08	41.5 ± 3.04	43.3 ± 10.8	45.4 ± 3.72^b^
TIBC	266.9 ± 7.5	279.8 ± 43.6	134.0 ± 52.6^f,a^	167.9 ± 52.0^b,c^

Results are shown as means ± SD

*WT_F* wild type female, *WT_M* wild type male, *H63D_F* H63D female, *H63D_M* H63D male, *Fe* iron, *Ts* transferrin saturation, *Hb* hemoglobin concentration, *RBC* red blood cells, *Ht* hematocrit, *TIBC* total iron-binding capacity

Statistical significant difference:

Fe; ^a^H63D_F/WT_F, *p* = 0.0320; ^b^H63D_M/WT_F, *p* = 0.0002; ^c^H63D_M/WT_M, *p* = 0.0002

Ts; ^b^H63D_M/WT_F, *p* = 0.0004; ^c^H63D_M/WT_M, *p* = 0.0004

Hb; ^d^H63D_F/H63D_M, *p* = 0.0002; ^b^H63D_M/WT_F, *p* = 0.0001; ^c^H63D_M/WT_M, *p* = 0.0006

RBC; ^d^H63D_F/H63D_M, *p* = 0.0005; ^b^H63D_M/WT_F, *p* = 0.0002; ^e^WT_F/WT_M, *p* = 0.0048

Ht; ^b^H63D_M/WT_F, *p* = 0.0124

TIBC; ^f^H63D_F/WT_M, *p* = 0.0001; ^a^H63D_F/WT_F, *p* = 0.0001; ^b^H63D_M/WT_F, *p* = 0.0001; ^c^H63D_M/WT_M, *p* = 0.0001

**Table 4:** The relationships between the iron (Fe) and hemoglobin (Hb) in control and H63D mutation groups

Group	Fe (μg/dl)	Hb (g/dl)	*R*	*p* value
WT	99.1 ± 30.7	13.8 ± 0.99	0.11	0.4368
H63D	151.5 ± 54.3	14.8 ± 1.47	0.44	0.0107^a^

Results are shown as means ± SD

*r* Pearson’s correlation coefficient, *WT_F* wild type female, *WT_M* wild type male, *H63D_F* H63D female, *H63D_M* H63D male

^a^Statistical significant difference

**Table 5:** The relationships between the iron (Fe) and hemoglobin (Hb) in various gender of control and H63D mutation groups

Group	Fe (μg/dl)	Hb (g/dl)	*R*	*p* value
WT_F	93.9 ± 26.8	13.4 ± 0.74	0.06	0.7832
WT_M	104.4 ± 33.9	14.2 ± 1.08	0.05	0.8225
H63D_F	134.0 ± 52.6	13.9 ± 0.90	0.12	0.6559
H63D_M	167.9 ± 52.0	15.7 ± 1.43	0.44	0.0595

Results are shown as means ± SD

*r* Pearson’s correlation coefficient, *WT_F* wild type female, *WT_M* wild type male, *H63D_F* H63D female, *H63D_M* H63D male

## Discussion

The impact of the HFE gene mutation on biochemical features of homozygotic and heterozygotic adults seems to be intelligible [2, 9–11, 14]. The diversity and frequency of gene mutation among populations provide a broad range of biochemical and occasionally clinical symptoms. However, the course of hemochromatosis is unpredictable since reduced gene penetration and possible concomitant environmental and epigenetic factors also play a role in disease development or progression [15, 16]. Therefore, knowing the HFE status of a child together with a precise analysis and follow-up of iron metabolism could identify an affected person who could potentially demonstrate clinical phenotype. In the current study, we compared iron metabolism of H63D HFE gene carriers, as it is the commonest in our population, with that of healthy children, with respect to gender. Statistical analysis displayed significantly higher iron, ferritin concentration, and transferrin saturation in H63D carrier group. Interestingly, all the parameters were higher in heterozygotes of both genders. This supports previous studies that involved HFE polymorphism and its biochemical impact on altered iron metabolism in childhood [17–19]. However, pathological iron loading in primary hemochromatosis is a prolonged and unpredictable process, affected by additional, innate and acquired conditions [1–4]. Results obtained in the present study suggest the need for genotyping chosen subjects from the developmental age. Moreover, an analysis performed on the association of iron and hemoglobin concentration revealed a positive correlation in HFE carrier group. Knowing the undoubted role of this trace element in hematopoiesis, our observations underline its unique impact on variant H63D carriers. The influence of unstable iron homeostasis on erythropoiesis modulation intensity by direct or indirect mediators of the metabolic pathways cannot be excluded [20, 21]. However, the HFE group presented with a positive iron-hemoglobin correlation, only male HFE carriers had statistically higher hemoglobin concentration compared to the other groups, presumably due to the direct effects of pubertal androgens [22].

Expertise on congenital hemochromatosis in children is limited; hence, recording and monitoring of any clinical or laboratory abnormalities in these probands seems to be justified.

## Conclusion

H63D variant of HFE gene increased iron absorption and present a positive impact on erythropoiesis modulation in children.
